# Supplementary material for: Single-cell transcriptomics and epigenomics unravel the role of monocytes in neuroblastoma bone marrow metastasis
Source: Nat Commun. 2023 Jun 26;14:3620. doi: 10.1038/s41467-023-39210-0 (PMC10293285; doi:10.1038/s41467-023-39210-0)
Supplement: Supplementary file 1 — Supplementary Information [file 41467_2023_39210_MOESM1_ESM.pdf]

SUPPLEMENTARY INFORMATION

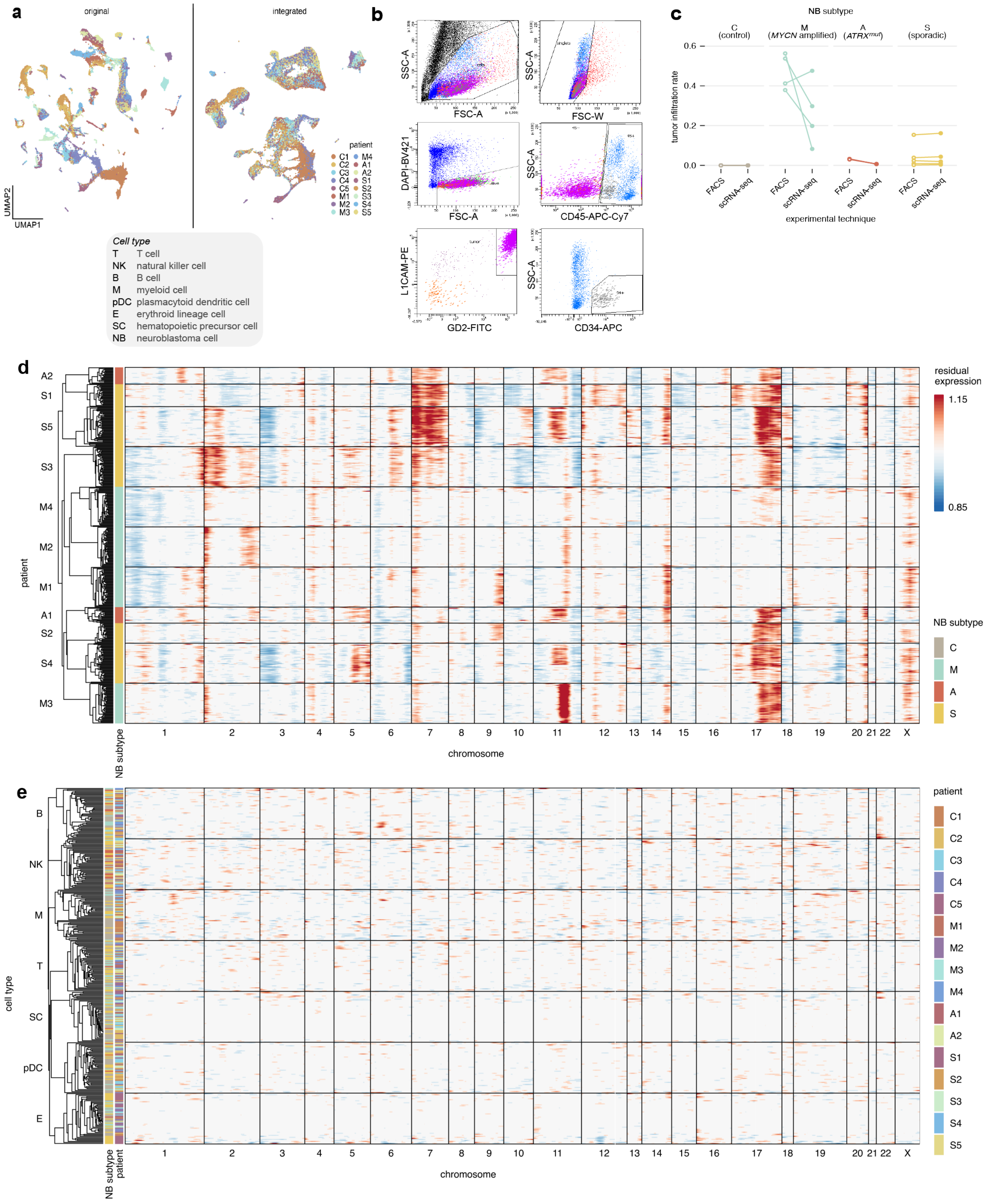

Supplementary Fig. 1. Cellular heterogeneity in BM samples of NB patients. (a) UMAP of the overall dataset before (left) and after (right) removal of patient effects. Each patient is colored-coded. (b-c) Gating

strategy for flow cytometry data and tumor infiltration rates in the NB subtypes as calculated from FACS data (CD45–/GD2+/L1CAM+ cells) and scRNA-seq data (the fraction of cells in cluster 8 as defined in Fig. 1b). (d) and (e) Analysis of copy number variation regions predicted from scRNA-seq data. (d) Residual gene expression in tumor cells after normalization relative to the microenvironment cells. (e) Residual gene expression in microenvironment cells after normalization relative to themselves. Genes (columns) in (d) and (e) are sorted by chromosome location. Source data are provided as a Source Data file.

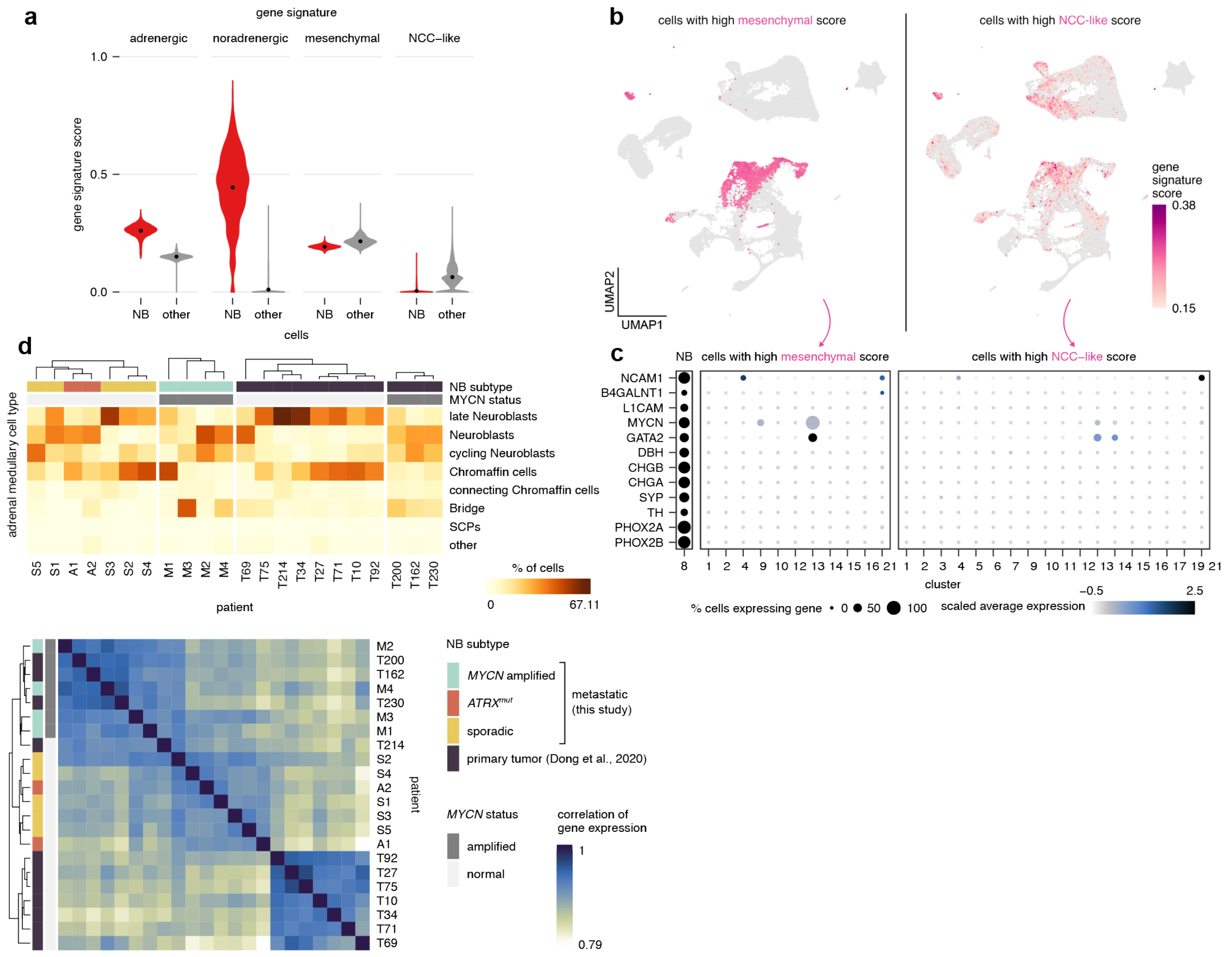

Supplementary Fig. 2. Inter- and intra-tumoral heterogeneity of metastatic NB cells. (a) Neuroblastoma-specific gene signature score distributions of tumor and microenvironment cells. (b) Locations of the top 5% cells with the highest mesenchymal (left) and NCC-like (right) gene signature scores on the UMAP of the overall dataset (n = 4,039 highlighted cells in each panel). (c) Expression of NB marker genes in a subset of the data that comprises the cells highlighted in (b) and all cells in cluster 8 (NB cells). (d) Relative abundance of the adrenal medullary cell types in our patient cohort (denoted with A: *ATRX*^{mut}, S: sporadic, M: MNA) and of Dong et al., [1] (denoted with T). Origin of tumor site (primary/metastasis) as well as MYCN amplification status shown in the bars above the heatmap. (e) Correlation analysis of pseudobulk gene expression demarcates the MNA tumors from *ATRX*^{mut} and sporadic tumors. Source data are provided as a Source Data file.

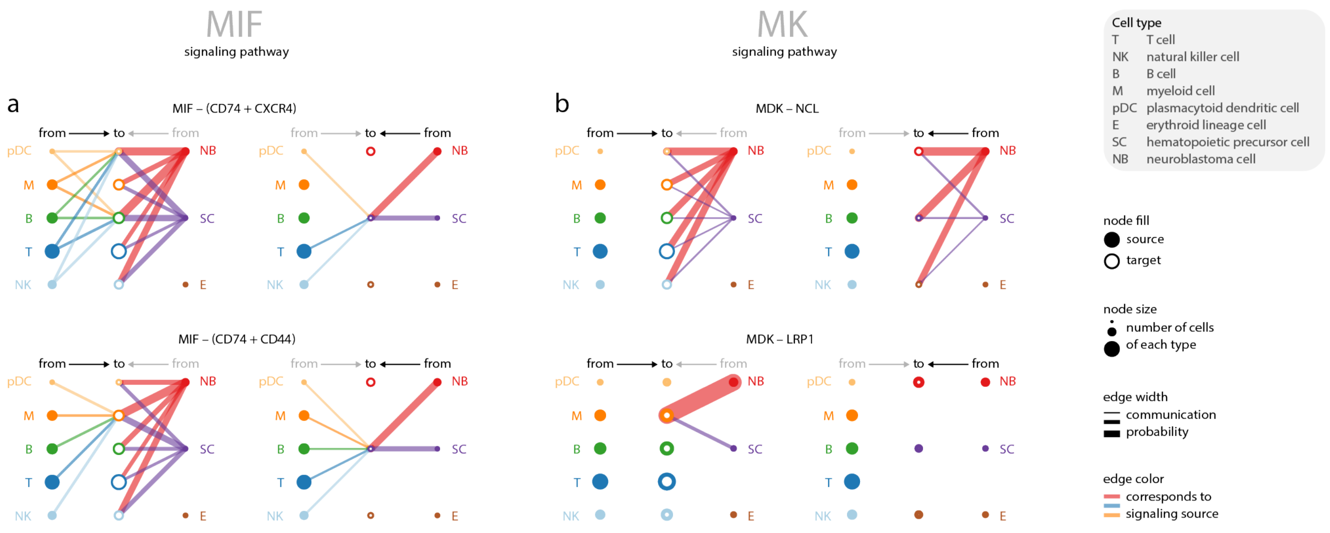

Supplementary Fig. 3. Interaction of NB cells with the local microenvironment. Inferred intercellular communication network for (a) macrophage inhibitory factor (MIF) and (b) midkine (MK) signaling. Source data are provided as a Source Data file.

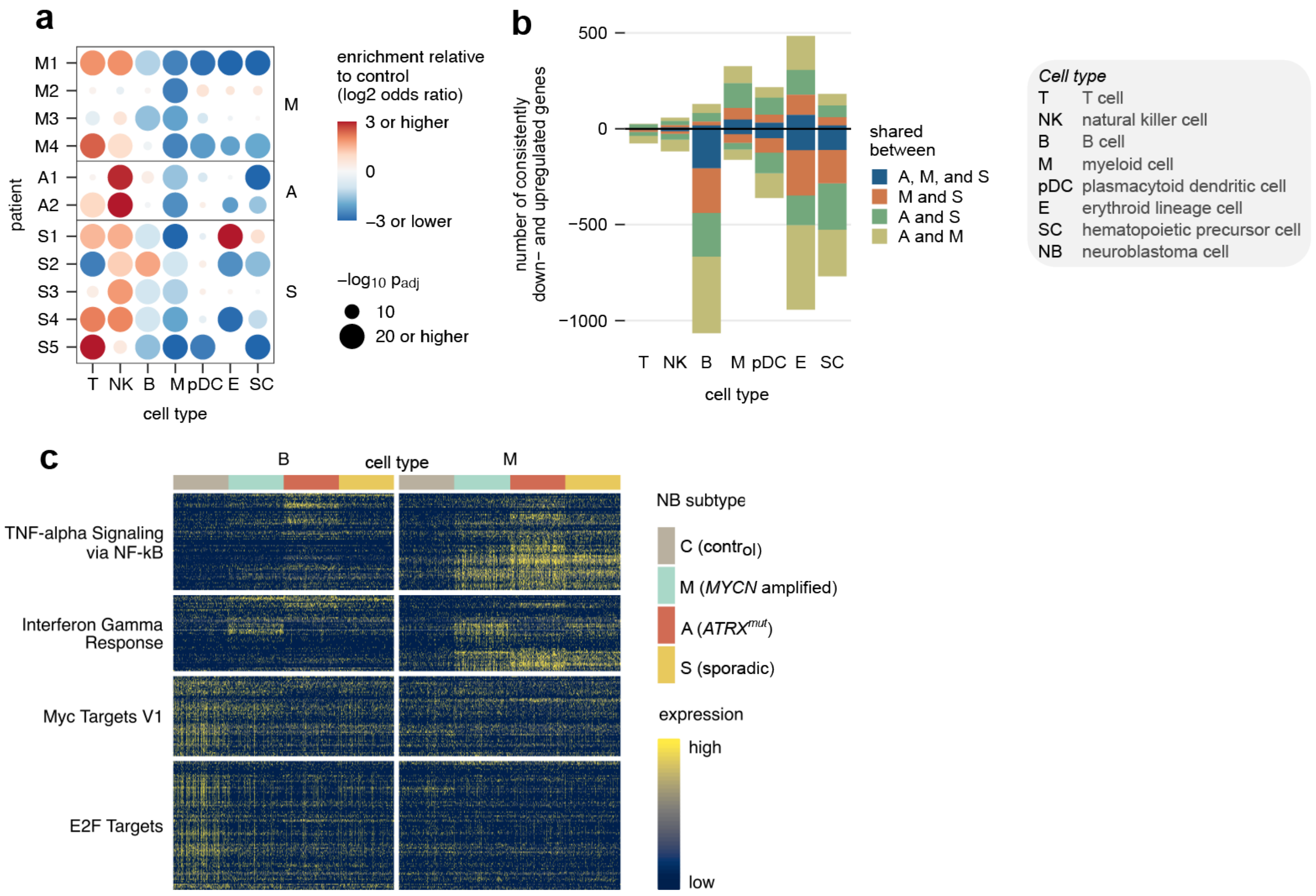

Supplementary Fig. 4. Cellular heterogeneity of NB-infiltrated BM compartment. (a) Enrichment of cell type abundance in individual NB patients compared to controls. Odds ratios and *p*-values were calculated by Fisher's exact test (two-sided). (b) Number of differentially expressed genes in NB subtypes compared to the controls. (c) Single-cell expression of exemplary genes in our patient cohort for the top two enriched and depleted pathways/targets identified in Fig. 4c. Source data are provided as a Source Data file.

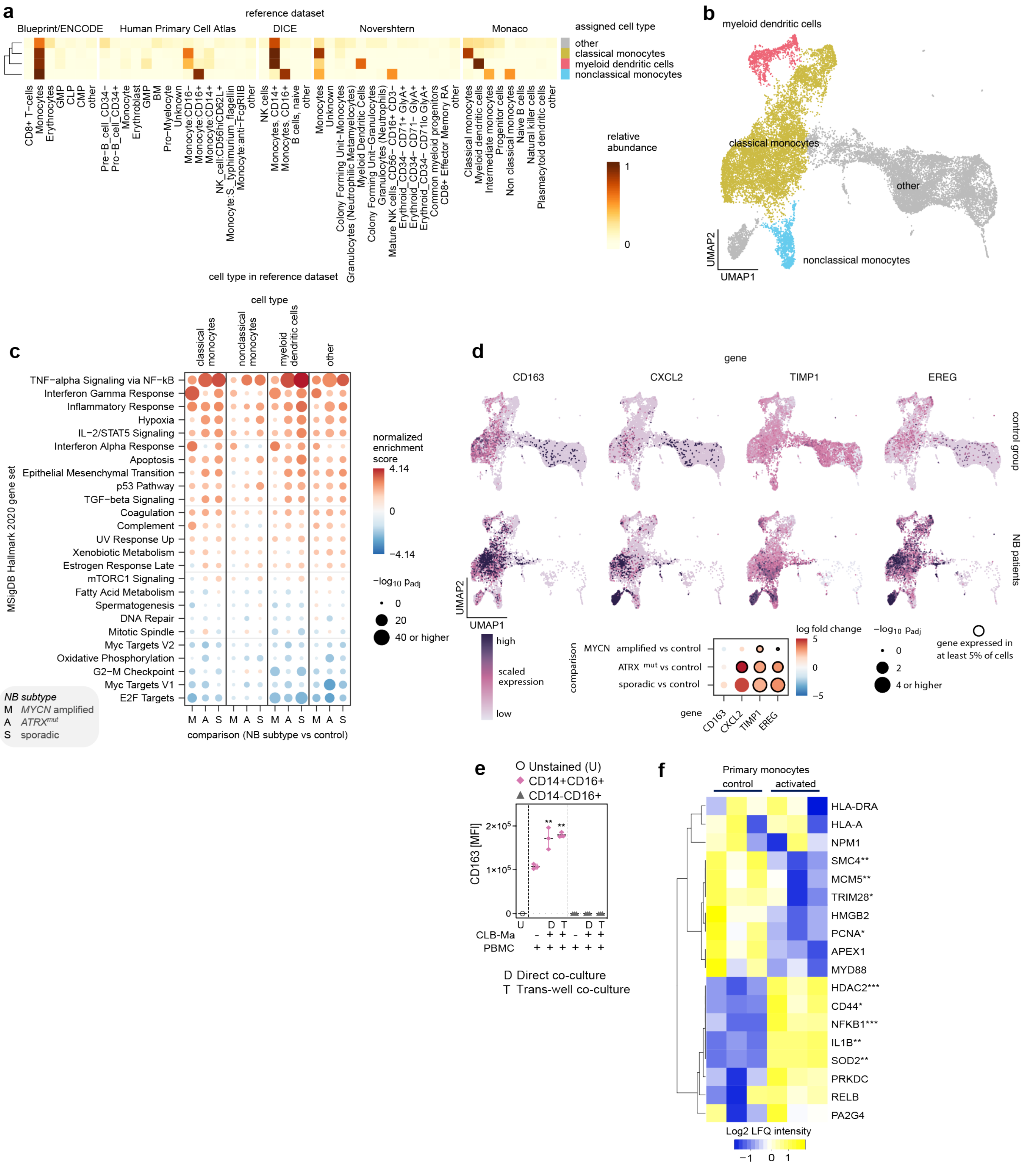

Supplementary Fig. 5. Characterization of the myeloid component within the BM compartment. (a) Relative abundances of cell types in each sub-cluster as predicted by comparing gene expression of each cell to gene expression in one of the reference datasets shown. The annotation to the right indicates the final cell type classification assigned to each cluster. (b) UMAP of myeloid cells (clusters 1, 2, 10, and 15 in Fig. 1b; n = 25,479 cells). Colors distinguish myeloid subtypes. (c) Gene set enrichment analysis using the MSigDB Hallmark 2020 database, with genes sorted according to their log-fold change of expression relative to the control group. *p*-values are based on an adaptive multi-level split Monte-Carlo scheme as implemented in the

R package fgsea. (d) Expression of selected myeloid and macrophage marker genes in controls and NB patients. *p*-values derive from gene-wise negative binomial mixed models using large-sample approximation as implemented in the R package nebula. (e) Mean fluorescence intensity of CD163 as determined by flow cytometry in NB cells, co-cultured either directly with PBMCs or through a trans-well for 3 days. Cells were gated for CD14+CD16+ and CD14-CD16+ populations (n=3 independent experiments). Data were subjected to one-way ANOVA and corrected using Dunnett's post hoc test for multiple comparisons (CD14+CD16+ population: PBMC vs. PBMC+CLB-Ma D, p=0.0033 and PBMC vs. PBMC+CLB-Ma TW, p=0.0018). (f) Label-free protein quantitation of targets identified in Fig. 4e in primary monocytes stimulated with LPS (n=3 independent experiments) and corresponding controls (n=3 independent experiments). Data were subjected to two-tailed paired Student's t-test and asterisks indicate statistically significant changes compared to respective controls: *p<0.05, **p<0.01, ***p<0.001. Data are presented as mean ± standard error of the mean (e). Source data are provided as a Source Data file.

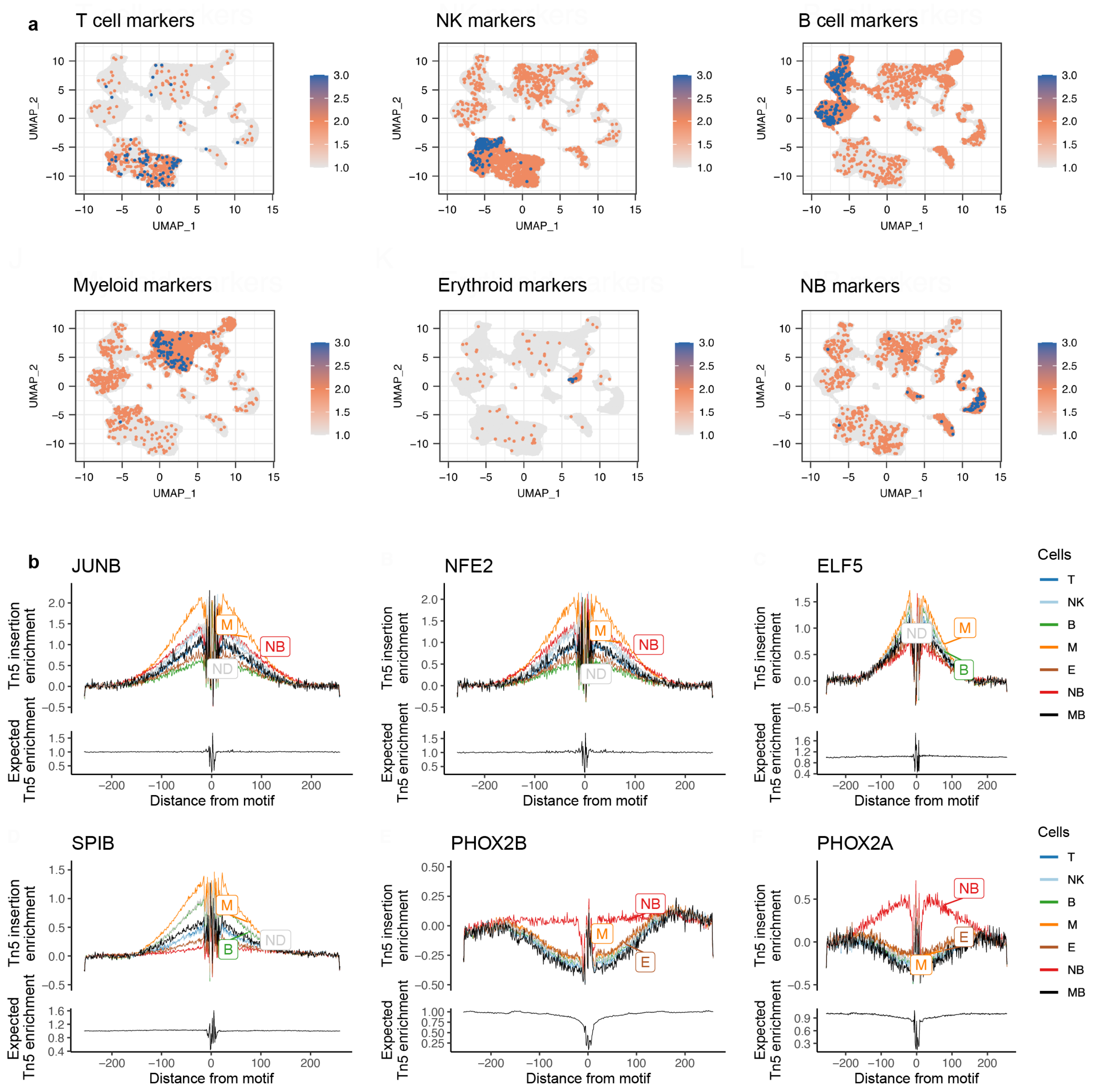

Supplementary Fig. 6. Single cell regulatory landscape of NB-infiltrated BM. (a) Combined marker genes signature score generated with Ucell mapped on to the UMAP generated with Signac (for details of the marker genes see online methods). (b) Enrichment of Tn5 integration events surrounding cell-type-specific TF motif sites with footprinting analysis. The Tn5 insertion bias tracks of corresponding TFs are shown above while the expected Tn5 enrichment profile is shown below. Source data are provided as a Source Data file.

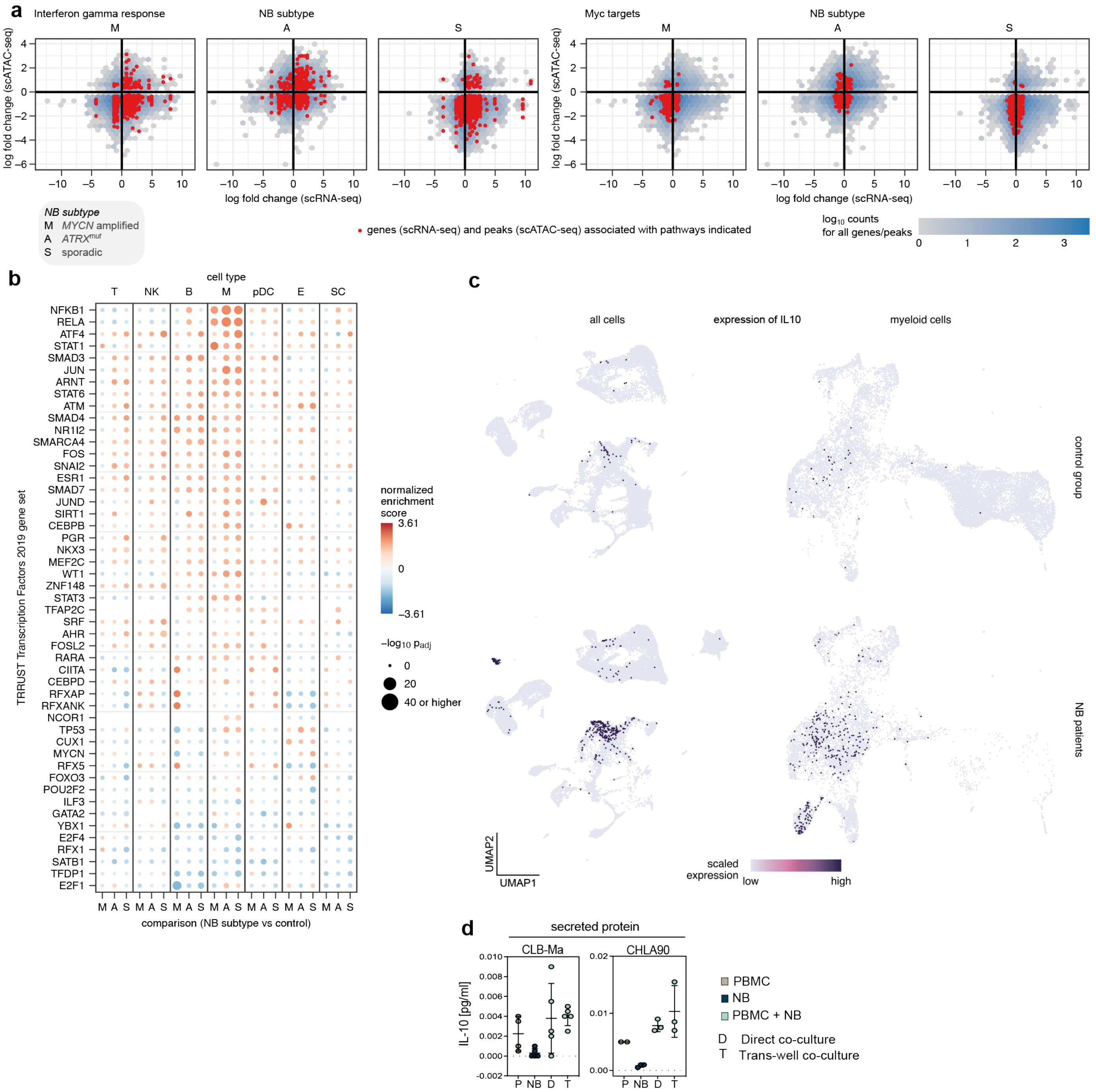

Supplementary Fig. 7. Transcriptional and epigenetic heterogeneity in myeloid cells in the BM compartment. (a) Scatterplot for genes in top enriched (IFNγ) and depleted pathways (MYC) between scRNA-seq and scATAC-seq log fold changes. (b) Gene set enrichment analysis using the TTRUST Transcription Factors 2019 database, with genes sorted according to their log-fold change of expression relative to the control group. p-values are based on an adaptive multi-level split Monte-Carlo scheme as implemented in the R package fgsea. (c) UMAP of the original clusters and the myeloid cell sub-clusters, respectively, showing the expression of IL10. (d) Secreted levels of IL10 as determined by ELISA in undiluted cell culture supernatants of CLB-Ma (n=4 independent experiments for PBMCs, n=5 independent experiments for all the other conditions) and CHLA90 (n=2 independent experiments for PBMCs, n=3 independent experiments for all the other conditions). Data are presented as mean ± standard error of the mean (d). Source data are provided as a Source Data file.

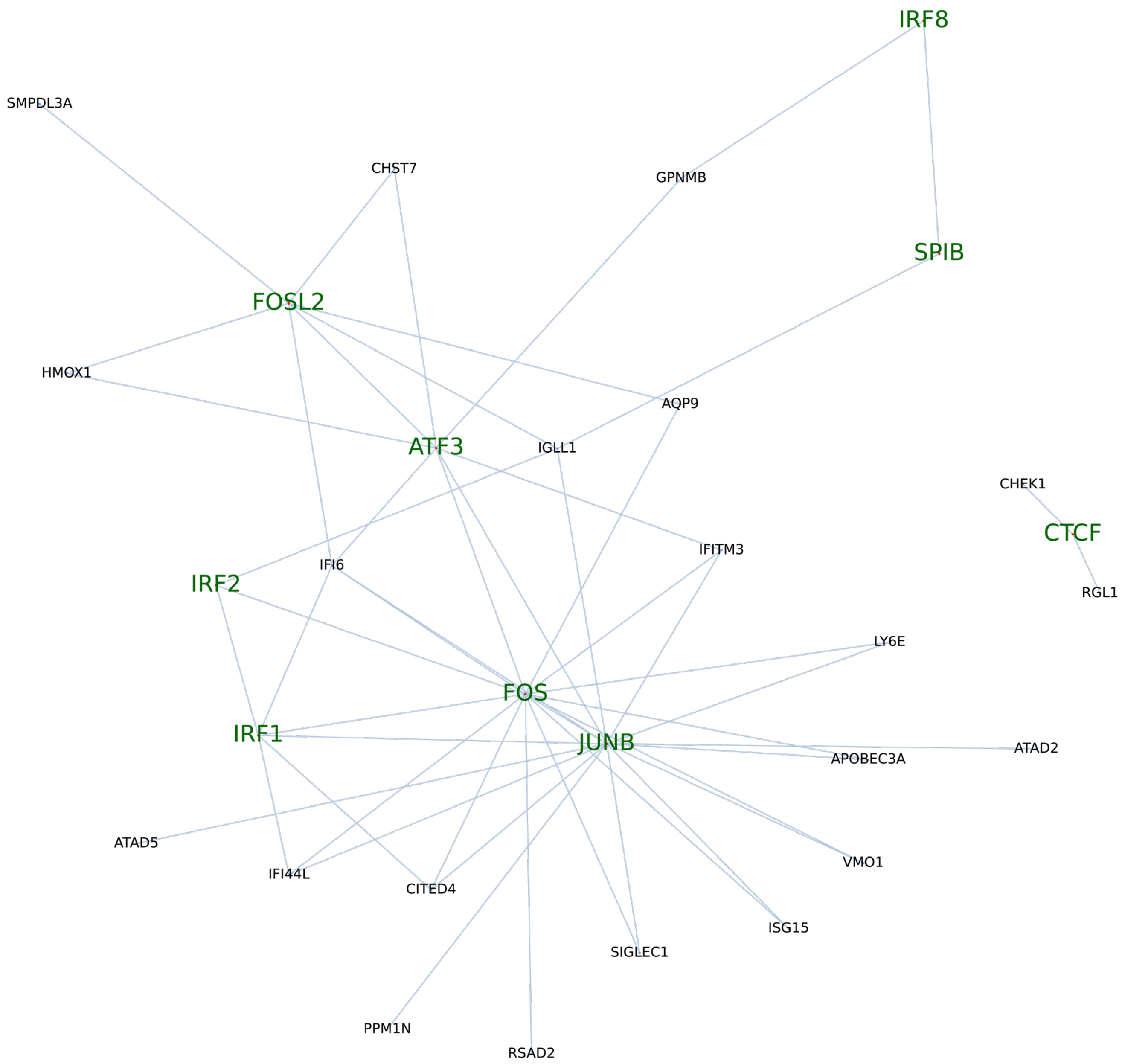

Supplementary Fig. 8. Regulatory sub-networks in myeloid cells. Transcription factors identified by motif analysis and their targets - differentially expressed genes based on comparisons of controls with *MYCN* amplified samples. Source data are provided as a Source Data file.

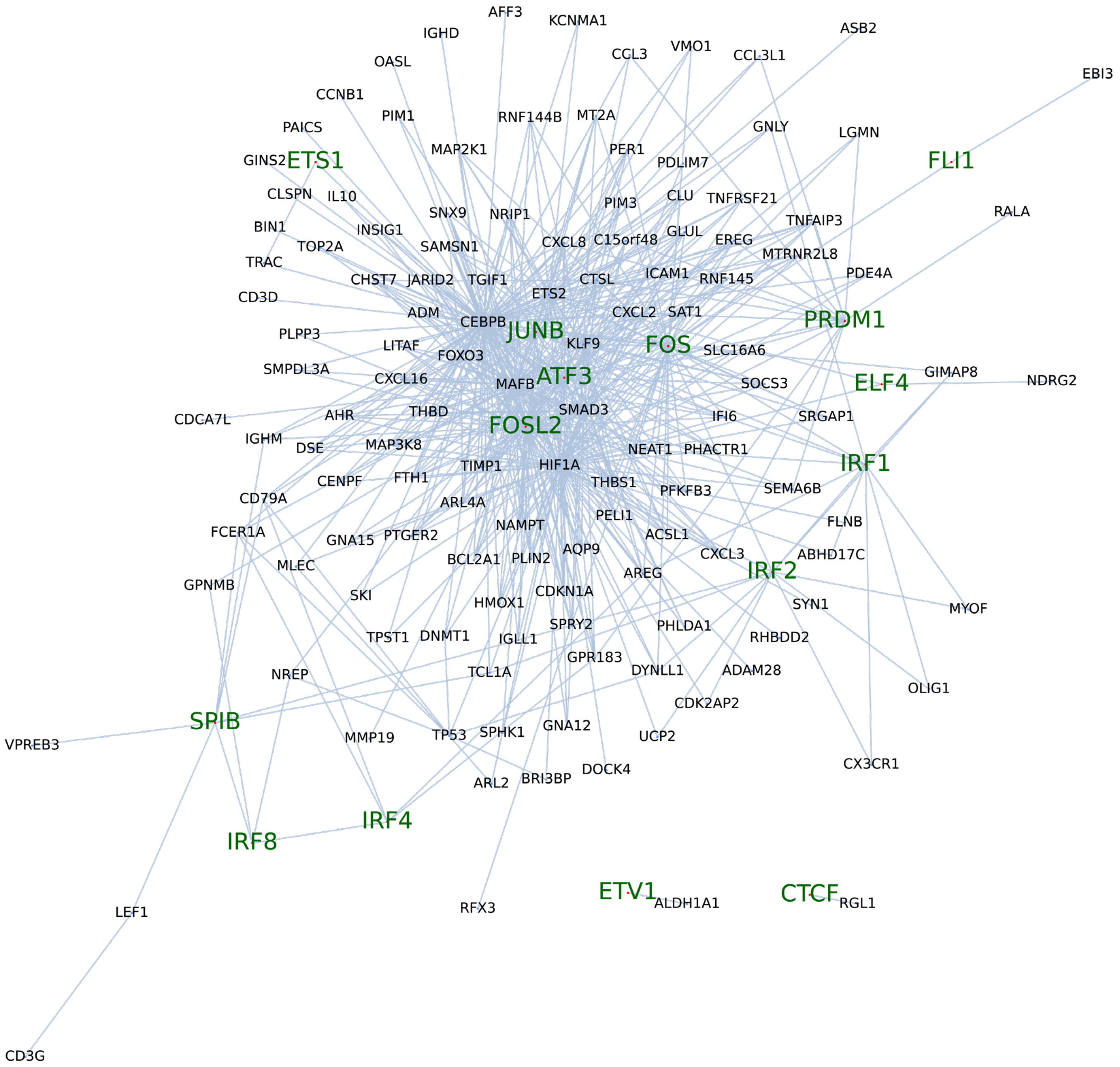

Supplementary Fig. 9. Regulatory sub-networks in myeloid cells. Transcription factors identified by motif analysis and their targets - differentially expressed genes based on comparisons of controls with *ATRX*^{mut} samples. Source data are provided as a Source Data file.

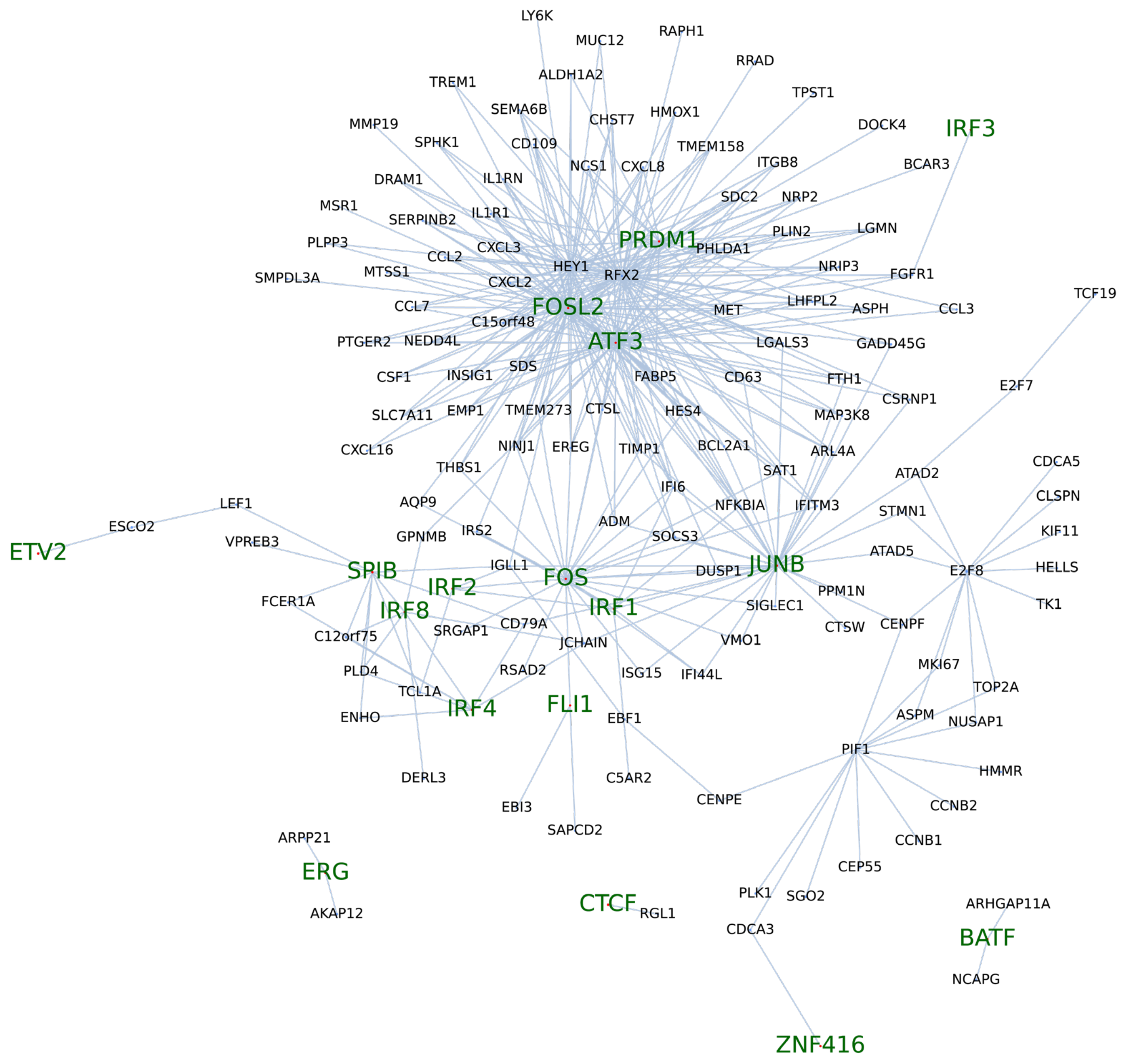

Supplementary Fig. 10. Regulatory sub-networks in myeloid cells. Transcription factors identified by motif analysis and their targets - differentially expressed genes based on comparisons of controls with sporadic samples. Source data are provided as a Source Data file.

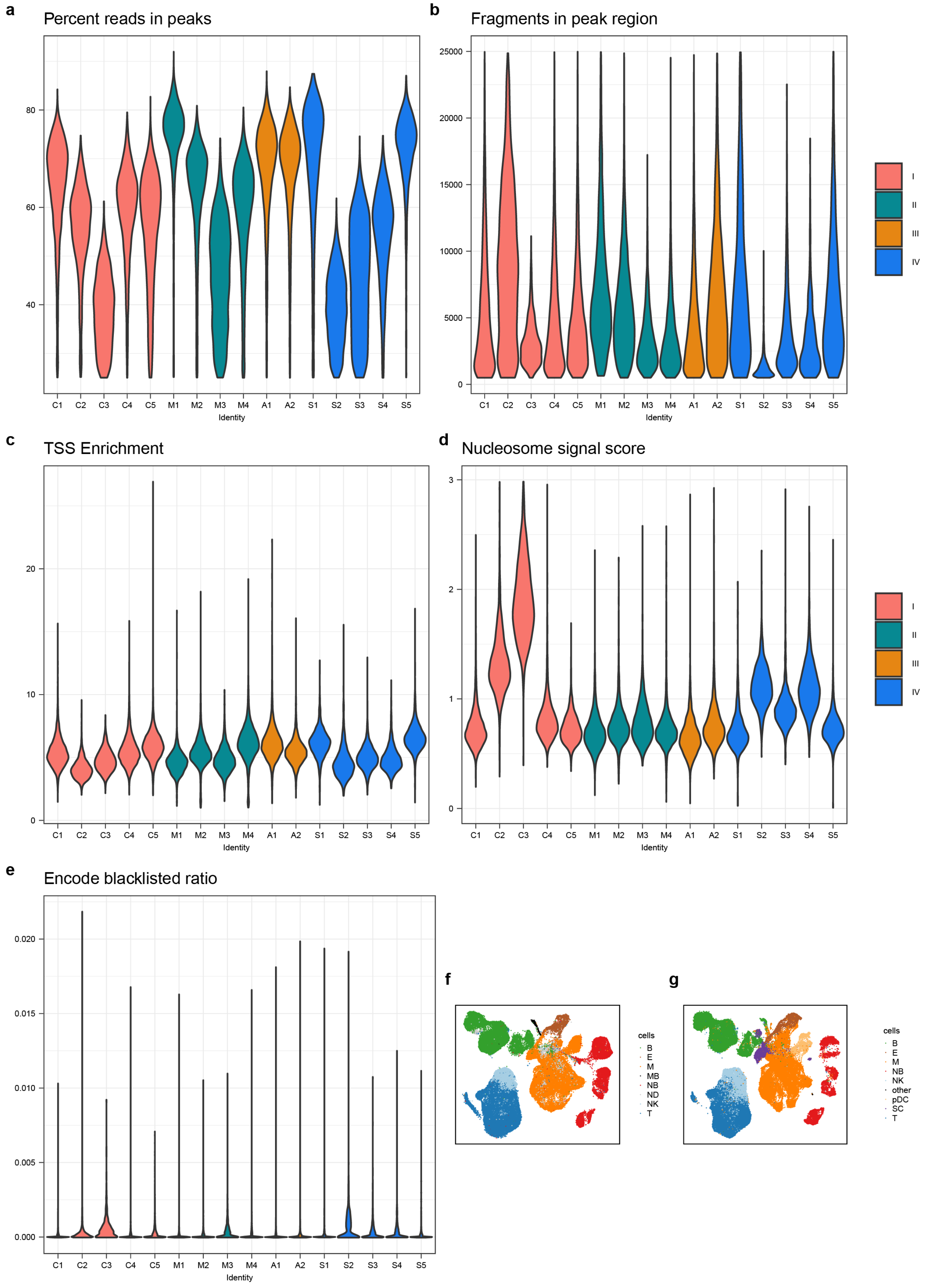

Supplementary Fig. 11. Quality control and integration of scRNA-seq and scATAC-seq.

(a-e) Quality control measures of scATAC-seq data. Cells with (a) at least 25% of reads in the peak region and (b) between 500 and 25000 fragments in peak region (c) with TSS enrichment score above 1, and (d) nucleosome signal score below 3 were selected for further analysis. (e) The fraction of encode blacklisted regions per sample is marginal. (f-g) Cells from scATAC-seq (f) and scRNA-seq (g) as well as their independently derived cell type annotations shown on to the integrated UMAP. Graph-based integration of scRNA-seq and scATAC-seq validates our cell-type annotations. Source data are provided as a Source Data file.

Supplementary Table 1. Summary of genetic and clinical characteristics

Sample ID	Pathology	Sex	Age at diagnosis >18mo	INSS stage	*MYCN*-amplification	*ATRX* intragenic deletion	Sample group	Tumor fraction by FACS [%]*
M1	NB	F	yes	4	yes	no	M	37.9
M2	NB	M	yes	4	yes	no	M	53.7
M3	NB	F	yes	4	yes	no	M	41.2
M4	NB	F	no	4	yes	no	M	56.3
A1	NB	M	yes	4	no	yes	A	3.3
A2	NB	M	yes	4	no	yes	A	3.0
S1	NB	M	yes	4	no	no	S	0.9
S2	NB	F	no	4	no	no	S	0.2
S3	NB	F	yes	4	no	no	S	15.4
S4	NB	F	yes	4	no	no	S	3.9
S5	NB	M	yes	4	no	no	S	2.4
C1	GNB	M	yes	n.a.	no	no	C	0
C2	GNB	F	yes	n.a.	no	no	C	0
C3	GN	M	yes	n.a.	no	no	C	0
C4	GN	M	yes	n.a.	no	no	C	0
C5	GN	F	yes	n.a.	no	no	C	0

* CD45-GD2+L1CAM+ cells of all cells alive.

Supplementary Table 2. Antibodies employed in FACS panels

**Patient samples**					
Antigen	Conjugate	Clone	Vendor	Catalog#	Dilution
CD34	APC	581	BD Biosciences	555824	1:200
CD45	APC-Cy7	2D1	BD Biosciences	561863	1:100
GD2	FITC	ch14:18	NA	NA	1:200
CD171	PE	REA163	Miltenyi	130-100-691	1:5
**Cell lines**					
Antigen	Conjugate	Clone	Vendor	Catalog#	Dilution
CD14	Alexa Flour 700	63D3	BioLegend	367114	1:50
CD16	BV605	3G8	BioLegend	302040	1:50
CD44	BUV737	G44-26	BD Biosciences	741840	1:50
CD45	PerCP	2D1	BD Biosciences	345809	1:25
CD74	BV711	LN2	BD Biosciences	743735	1:25
CD86	BV510	2331	BD Biosciences	563461	1:50
CD163	PE	MAC2-158	BD Biosciences	567881	1:350
CD171	PE	REA163	Miltenyi	130-100-691	1:8
CXCR4	PE-Cy5	12G5	BD Biosciences	555975	1:20
HLA-ABC	PE-Cy5	G46-2.6	BD Biosciences	555554	1:100
HLA-DR	BV510	L243	BioLegend	307646	1:50
GD2	FITC	ch14:18	NA	NA	1:1000
LRP1	BUV805	A2MR-α2	BD Biosciences	748694	1:25
NCL	Alexa Flour 647	NCL/902	Novus Biologicals	NBP2-47860AF647	1:25

NA, not available

REFERENCES

1. Dong, R., et al., *Single-Cell Characterization of Malignant Phenotypes and Developmental Trajectories of Adrenal Neuroblastoma.* Cancer Cell, 2020. **38**(5): p. 716-733.e6.
