# Supplementary material for: Single-cell transcriptomics and epigenomics unravel the role of monocytes in neuroblastoma bone marrow metastasis
Source: Nat Commun. 2023 Jun 26;14:3620. doi: 10.1038/s41467-023-39210-0 (PMC10293285; doi:10.1038/s41467-023-39210-0)
Supplement: Supplementary file 4 — Description of Additional Supplementary Files [file 41467_2023_39210_MOESM4_ESM.pdf]

**Description of Additional Supplementary Files**

Supplementary Data 1
Description: scRNA sequencing statistics

Supplementary Data 2
Description: Copy number variation regions as predicted from scRNA-seq data

Supplementary Data 3
Description: Cell-cell interactions. p-values are computed from a one-sided permutation test as implemented in the R package CellChat

Supplementary Data 4
Description: Differentially expressed genes in the overall dataset. p-values derive from gene-wise negative binomial mixed models using large-sample approximation as implemented in the R package nebula

Supplementary Data 5
Description: Gene set enrichment analysis results for the overall dataset. p-values are based on an adaptive multi-level split Monte-Carlo scheme as implemented in the R package fgsea

Supplementary Data 6
Description: Gene set enrichment analysis results for myeloid cells. p-values are based on an adaptive multi-level split Monte-Carlo scheme as implemented in the R package fgsea

Supplementary Data 7
Description: Differentially expressed genes in myeloid cells. p-values derive from gene-wise negative binomial mixed models using large-sample approximation as implemented in the R package nebula

Supplementary Data 8
Description: scATAC sequencing statistics
